# Extraction of Copper from Sulfuric Acid Solutions Based on Pseudo-Liquid Membrane Technology

**DOI:** 10.3390/membranes13040418

**Published:** 2023-04-07

**Authors:** Artak E. Kostanyan, Vera V. Belova, Yulia A. Zakhodyaeva, Andrey A. Voshkin

**Affiliations:** Kurnakov Institute of General and Inorganic Chemistry, Russian Academy of Sciences, 31 Leninskii pr., 119991 Moscow, Russia

**Keywords:** liquid and pseudo-liquid membranes, three-phase extractors, copper extraction from sulfuric acid solutions

## Abstract

Pseudo-liquid membranes are extraction devices in which a liquid membrane phase is retained in an apparatus consisting of two interconnected chambers while feed and stripping phases pass through the stationary liquid membrane phase as mobile phases. The organic phase of the liquid membrane sequentially contacts the aqueous phases of the feed and stripping solutions in the extraction and stripping chambers, recirculating between them. This extraction separation method, called multiphase pseudo-liquid membrane extraction, can be implemented using traditional extraction equipment: extraction columns and mixer-settlers. In the first case, the three-phase extraction apparatus consists of two extraction columns connected at the top and bottom by recirculation tubes. In the second case, the three-phase apparatus consists of a recycling close-loop, which includes two mixer-settler extractors. In this study, the extraction of copper from sulfuric acid solutions in two-column three-phase extractors was experimentally studied. A 20% solution of LIX-84 in dodecane was used as the membrane phase in the experiments. It was shown that the extraction of copper from sulfuric acid solutions in the apparatuses studied was controlled by the interfacial area in the extraction chamber. The possibility of the purification of sulfuric acid wastewaters from copper using three-phase extractors is shown. To increase the degree of extraction of metal ions, it is proposed to equip two-column three-phase extractors with perforated vibrating discs. To further increase the efficiency of extraction using the pseudo-liquid membrane method, it is proposed to use multistage processes. The mathematical description of multistage three-phase pseudo-liquid membrane extraction is discussed.

## 1. Introduction

Bulk, supported, emulsion, and pseudo-liquid membrane separations allow two-stage separation of the components of an aqueous solution, combining the extraction and re-extraction (stripping) processes in one technological operation. In all types of liquid membranes, the separation of the components is based on their distribution between two aqueous phases through an organic transfer medium (liquid membrane). One of the simplest types of liquid membranes is the bulk liquid membrane [1,2,3,4,5,6,7,8,9], but its disadvantage—low mass transfer efficiency caused by small interfacial area per unit volume—makes it unpromising for large-scale industrial applications. In supported liquid membranes [10,11,12,13,14,15,16,17,18,19], two aqueous phases (feed and stripping phases) flow on opposite sides of the porous support in the small pores of which the liquid membrane is retained. In the emulsion liquid membrane technique [20,21,22,23,24,25,26,27,28,29,30,31,32,33,34], complex water-in-oil emulsions are used: the first aqueous (stripping) phase is encapsulated in the form of microdroplets in the organic membrane; large droplets fall or rise in the continuous second aqueous phase (the feed solution or raffinate phase). In pseudo-liquid membrane separation, the process is carried out in a three-phase mass transfer stage (extraction apparatus), consisting of two interconnected contact chambers in which the membrane phase contacts the feed and stripping phases and recirculates inside the stage, forming a closed circuit [35,36].

Compared to traditional liquid extraction, the main advantages of liquid membrane separation are significant savings in reagents and solvents, as well as better separation of mixture components, especially when processing dilute solutions. In addition, liquid membrane methods are more environmentally friendly since the organic phase is not removed from the process unit. Therefore, liquid membrane methods can be used to separate and concentrate metal ions from dilute aqueous solutions when solvent extraction processes cannot be effectively applied. Due to the low productivity and complexity of the equipment, traditional methods of liquid membrane separation have not yet found wide industrial applications. Since the currently available liquid membrane equipment cannot process large volumes of dilute solutions, we previously [37,38,39,40,41,42,43,44,45] proposed the use of solvent extraction equipment as a large-scale device for bulk liquid membrane separation processes. This bulk liquid membrane separation method based on the extraction equipment has been called three- and multiphase extraction or pseudo-liquid membrane multiphase extraction [46]. It may also be referred to as pseudo-liquid membrane technology. This name (more precisely, electrostatic pseudo-liquid membranes) was apparently first introduced by Chinese researchers [35,36] to designate a device filled with a non-polar transport medium through which two dispersed aqueous phases are passed: the feed solution containing metal ions and the receiver phase. Drops of both aqueous phases break up into smaller droplets under the influence of a high-voltage electrostatic field; flows of dispersed phases are separated by a partition [47,48]. With the help of strong electrostatic fields, it is possible either to separate water droplets from the organic phase by electrocoalescence or to deform and break the droplets into smaller ones. The work of [49] presents the results of an experimental investigation of drop break-up in different liquid–liquid systems. In [47], the separation of nickel and cobalt in an electrostatic pseudo-liquid membrane apparatus was investigated using a pregnant leach solution as a feed solution. The effects of operating parameters, such as feed and strip flow rates, pH, and applied voltage, have been studied. In [50], electrospun hybrid membranes were applied to remove heavy metals and produce ultra-pure water. It was established that the introduction of a condensed Ti-O-Ti structure into the membranes significantly increased the performance but did not improve the permeate flux of the membranes. Asymmetric thermal treatment of the membranes resulted in an increase in the permeate flux without affecting the separation.

**Figure 1:**
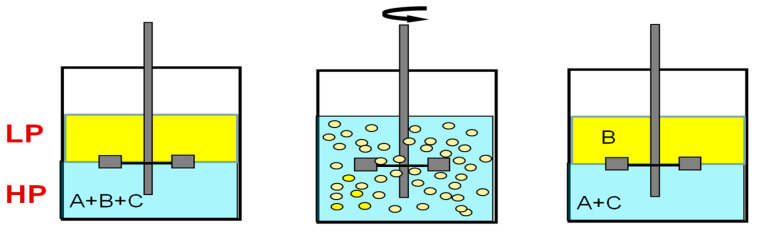
General scheme of extraction processes. A–C—components of the mixture.

In the processes of pseudo-liquid membrane separation and extraction separation, first, one of the phases (light or heavy) is dispersed in the form of droplets in the second phase due to intensive mixing, and then droplet coalescence and gravitational phase separation occur (Figure 1).

**Figure 2:**
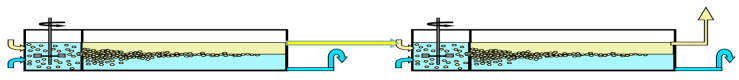
Two mixer-settlers operating in pseudo-liquid membrane mode.

Solvent extraction technologies use mixer-settlers and column extractors to carry out these processes. Mixer-settler extractors are widely used in hydrometallurgy to separate metal compounds, including rare earth elements. A mixer-settler extractor consists of two chambers: a mixing chamber, where the organic and aqueous phases are mixed to form an emulsion, and a separation chamber (settler), where the emulsion is separated. Figure 2 is a diagram of the connection of two mixer-settlers operating in pseudo-liquid membrane mode. The organic membrane phase circulates between the extractors, sequentially contacting the aqueous phases of the feed and stripping solutions in the extraction and stripping apparatuses. In such a system, the membrane phase can be either dispersed or continuous in the mixing chambers of the extractors.

Column extractors are used in the chemical industry for the separation and purification of organic and inorganic compounds from process solutions and industrial wastewater treatment. These apparatuses can be divided into two groups: (1) columns with an external energy input, in which kinetic energy is supplied with agitators of various types for dispersion of one of the phases (dispersed) in the volume of the other (continuous) and (2) columns without energy input. In the apparatuses of the first group, sufficient interfacial area is provided, and interphase mass transfer is enhanced by the energy supply.

Similar to the combination of two mixer-settlers, two columns (for extraction and stripping) can be connected at the top and bottom, with overflows for circulation of the membrane phase to create a two-chamber three-phase extractor operating in pseudo-liquid membrane mode. In such a system, for convective circulation of the membrane phase, this phase should be continuous in the columns of the three-phase extractor. Convective circulation of the membrane phase between the extraction and stripping columns can be organized due to the difference in the weight of the emulsion in the columns through which the dispersed aqueous feed and stripping phases pass.

**Figure 3:**
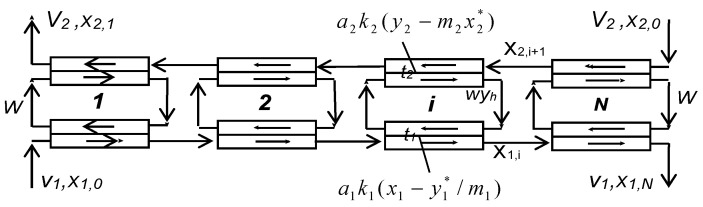
Schematic diagram of multistage three-phase extraction processes.

Multistage processes are commonly used to enhance extraction separation processes. The mathematical models of various schemes of multistage pseudo-liquid membrane extraction were developed in our previous studies [39,40,46,51,52,53]. The theoretical model of multistage two-column pseudo-liquid membrane extraction with countercurrent flows of phases in extraction and stripping columns is shown in Figure 3. To simulate such extraction separations, the following equations can be used:

(1) $$\frac{x_{1,N}}{x_{1,0}}=\frac{1-F_{1}F_{2}}{{\left[\frac{1-\varphi }{1-\varphi /\left(F_{1}F_{2}\right)}\right]}^{N}-F_{1}F_{2}}$$

(2) $$\varphi =\frac{F_{1}F_{2}S_{1}S_{2}}{S_{2}+F_{1}S_{1}\left(1-S_{2}\right)}$$

(3) $$S_{1}=\frac{1-exp\left[t_{1}\left(F_{1}-1\right)\right]}{1-F_{1}\;exp\left[t_{1}\left(F_{1}-1\right)\right]}$$

(4) $$S_{2}=\frac{1-exp\left[t_{2}\left(F_{2}-1\right)\right]}{1-F_{2}\;exp\left[t_{2}\left(F_{2}-1\right)\right]}$$

where $F_{1}=v_{1}/\left(wm_{1}\right)$ and $F_{2}=wm_{2}/v_{2}\;$ are dimensionless parameters (extraction and stripping factors); $t_{1}=a_{1}k_{1}L_{1}/v_{1}$ and $t_{2}=a_{2}k_{2}L_{2}/w$ are dimensionless parameters characterizing the mass transfer rate in extraction and stripping columns, known as the numbers of transfer units; *L_1_* and *L_2_* are the column lengths; $x_{1,0}$ and $x_{1,N}$ are the concentrations of a solute in the feed and in the raffinate; $a_{1}$ and $a_{2}$ are specific phase contact surfaces in the extraction and stripping columns; $k_{1}$ and $k_{2}\;$ are mass-transfer coefficients at the extraction and stripping steps, respectively; $v_{1}$ and $v_{2}$ are specific flow rates of the feed and raffinate phases; *w* is the specific flow rate (circulation rate) of the membrane phase; and $x_{1}$ and $x_{2}$ are the concentrations in the feed and raffinate phases (symbol * denotes equilibrium conditions).

Equations (1)–(4) are based on the assumptions that equilibrium-distribution coefficients $m_{1}=y^{*}/x_{1}^{*}$ and $m_{2}=y^{*}/x_{2}^{*}$ are constant and that the phases in the extraction and stripping columns move countercurrently and in the plug-flow mode. When the pseudo-liquid membrane multistage extraction processes are carried out in a cascade of mixer-settler extractors, equilibrium distribution of the passing components is achieved in the extraction and stripping chambers, and they represent theoretical stages (*t*_1_→∞, *t*_2_→∞). The concentration in the raffinate in these processes can be calculated using the following equation:

(5) $$\frac{x_{1,N}}{x_{1,0}}=\frac{1-F_{1}F_{2}}{{\left[\frac{1+F_{1}}{\left(1+F_{2}\right)F_{1}}\right]}^{N}-F_{1}F_{2}}$$

As the rate of membrane phase circulation between the extraction and stripping steps rises (*w*→∞), the concentration ratio in the phases in the extraction and stripping apparatuses approaches an equilibrium value equal to *m*_1_/*m*_2_, and Equation (5) reduces to

(6) $$\frac{x_{1,N}}{x_{1,0}}=\frac{F^{N}-F^{N+1}}{1-F^{N+1}}$$

with

(7) $$F=\frac{v_{1}m_{2}}{v_{2}m_{1}}$$

In conventional countercurrent extraction in a cascade of *N* equilibrium stages, the concentration in the raffinate is determined by Equation (8):

(8) $$\frac{x_{N}}{x_{0}}=\frac{f^{N}-f^{N+1}}{1-f^{N+1}}$$

where

$f=\frac{v_{x}}{v_{y}m}$ is the extraction factor; $y^{*}=mx^{*}$.

From Equations (6) and (8), the conclusion can be drawn that Equation (6) determines the efficiency of the pseudo-liquid membrane multistage extraction carried out in a cascade of *N* theoretical two-chamber mass-transfer stages.

In our recently published work [37], we presented the results of an experimental study of the extraction of phenol from sulfuric acid solutions in two-column pseudo-liquid membrane apparatuses using butyl acetate as an organic membrane phase and a 5–12% NaOH aqueous solution as a stripping phase. It was shown that the devices under consideration can be successfully used for wastewater treatment from phenol and multiple concentrations of phenolate in the stripping phase, which can be achieved due to the recirculation of this phase. Earlier, in [38], the possibility of successful separation of uranyl, ytterbium, and lanthanum nitrates in a three-column multiphase extractor was established.

In this work, the results of an experimental study of the extraction of copper in two-column pseudo-liquid membrane extractors are presented. The purpose of the copper extraction experiments was to study the possibility of the purification of sulfuric acid wastewaters generated in the production of non-ferrous metals and in the processing of products from copper using two-column pseudo-liquid membrane extractors.

## 2. Materials and Methods

### 2.1. Reagents

The following reagents were used in this study: copper(II) sulfate pentahydrate (98%, CAS no. 7758-99-8, Sigma-Aldrich, St. Louis, MO, USA), LIX-84 (BASF, Indonesia), dodecane (CAS no. 13475-82-6, Acros Organics, Germany), decane (99+%, pure, CAS no. 124-18-5, Acros Organics, Germany), n-undecane (99%, CAS no. 1120-21-4, Acros Organics, Germany), and sulfuric acid (chemically pure, Khimreaktivsnab, Russia).

### 2.2. Apparatuses

As mentioned above, a pseudo-liquid membrane device is a three-phase extractor comprising an extraction column and a stripping column, each of which consists of contacting and separation zones; the columns are connected by overflows for circulation of a continuous membrane phase. In the extraction column, the feed solution is brought into contact with the membrane phase, from which the extracted compounds are stripped in the second column with a stripping phase. The feed solution and the stripping phase are each dispersed into droplets in the appropriate column by means of dispersing devices. Owing to the difference in density between the emulsions in the first and second columns, circulation of the continuous phase, which is the extractant phase, occurs through the upper and lower overflows, resulting in transfer of the substances to be extracted from one column to the other and from the first dispersed phase into the second.

**Figure 4:**
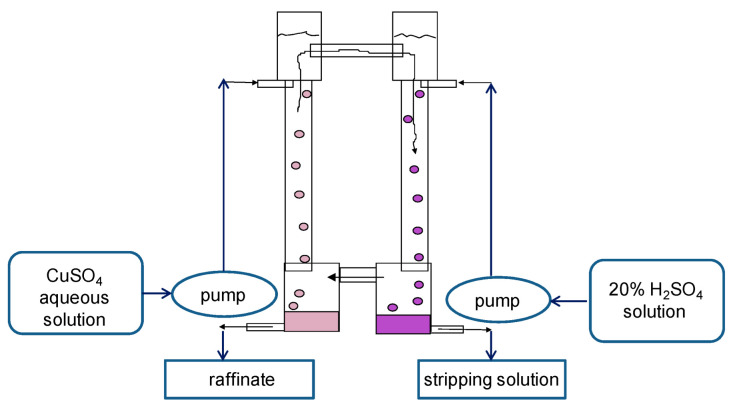
Scheme of the experimental setup of the small two-column extraction apparatus. Columns have a diameter of 30 mm and a height of 400 mm. To extract copper from sulfuric acid solution, a 20% solution of LIX-84 in dodecane was used as an organic membrane phase, and a 20% solution of sulfuric acid was used as a stripping phase.

**Figure 5:**
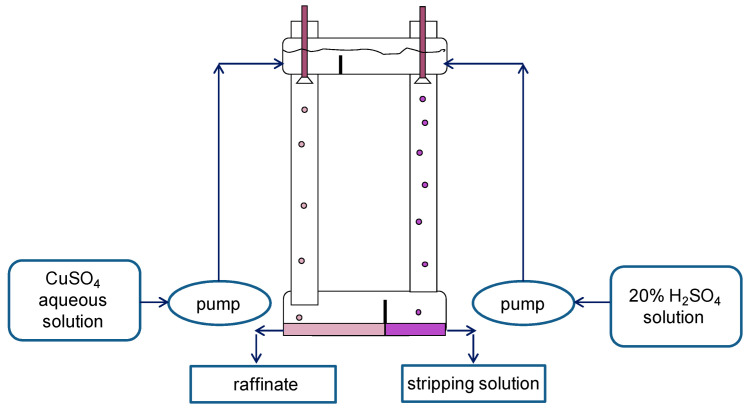
Scheme of the experimental setup of the large two-column extractor. Columns have a diameter of 80 mm and a height of 900 mm. To extract copper from sulfuric acid solution, 20% LIX-84 was used as an organic phase in a mixture containing 42% dodecane, 31% decane, and 27% undecane, and a 20% solution of sulfuric acid was used as a stripping phase.

For experimental studies, two two-column glass apparatuses were designed and manufactured: (1) a small one: columns with a diameter of 30 mm and a height of 400 mm in which the columns are connected by overflow pipes and equipped with bottom settlers and upper separating zones (Figure 4); and (2) a large two-column extractor whose columns have a diameter of 80 mm and a height 900 mm; the columns are connected at the bottom (by a horizontal cylindrical settler 80 mm in diameter) and at the top (by a separator of a similar design) (Figure 5). For the dispersion of aqueous phases in the columns, fluoroplastic distributors with different hole diameters were used.

### 2.3. Feed Solutions and Extraction Systems

To extract copper from sulfuric acid solutions, the 20% solution of LIX-84 in dodecane was used as the organic membrane and the 20% sulfuric acid solution as the stripping phase. The concentration of copper was determined in aqueous solution—by the photometric method in the form of an ammonia complex—and in the organic phase—from the difference between the concentrations in the feed solution and in the aqueous phase after extraction.

## 3. Results and Discussion

The equilibrium distribution of copper from sulfuric acid solution in the system with 20% LIX-84 in decane was preliminarily studied. The initial aqueous solution contained 1225 ppm CuSO_4_. In the experiments, the ratio of the aqueous and organic phases varied. The data obtained are presented in Table 1.

The results of the experiments on apparatus 1 are shown in Table 2. As can be seen from the data in Table 2, when using distributors with smaller diameter holes, the extraction of copper increases. In these experiments, the cocurrent flow of the phases in the extraction column and the countercurrent flow of the aqueous and organic phases in the stripping column were observed.

In experiments on the large three-phase extractor, 20% LIX-84 was used as the organic phase in a mixture containing 42% dodecane, 31% decane, and 27% undecane. Two series of experiments were carried out in this apparatus. In the first series, as in apparatus 1, the crushing of aqueous phases was carried out using an inlet dispersing device (distributor). The results of this series of experiments are shown in Table 3, from which it follows that the extraction of copper increased slightly due to the increase in the height of the columns. The low efficiency of the apparatuses is due to the fact that the dispersing devices do not provide a sufficiently large phase contact surface (large droplets are formed during crushing by distributors). Distributors located at the top of the columns allowed only a single crushing of the aqueous phases, while agitators provided more intensive multiple crushing with the formation of small droplets.

To intensify dispersion in subsequent experiments, nine perforated discs (sieve plates) made of PTFE were placed in the extraction column. The discs were mounted on a vertical rod connected to a vibration drive. The discs were given high-frequency oscillations (50 sec^−1^) with an amplitude of 1 mm. As follows from Table 4, the efficiency of the process increased dramatically.

In experiments 4 and 5, the oscillation amplitude was increased by 20%, which decreased the copper concentration in the raffinate by another order of magnitude. We can conclude that the interphase surface in the extraction column determines the efficiency of copper extraction from sulfuric acid solutions in the two-column extractor. It is known that the mass transfer rate in extraction is proportional to the interfacial area per unit volume of the contacting phases. In addition, the extraction of metal ions is usually associated with a chemical reaction at the phase interface. Therefore, an increase in the phase contact surface leads to an intensification of the metal extraction processes.

**Figure 6:**
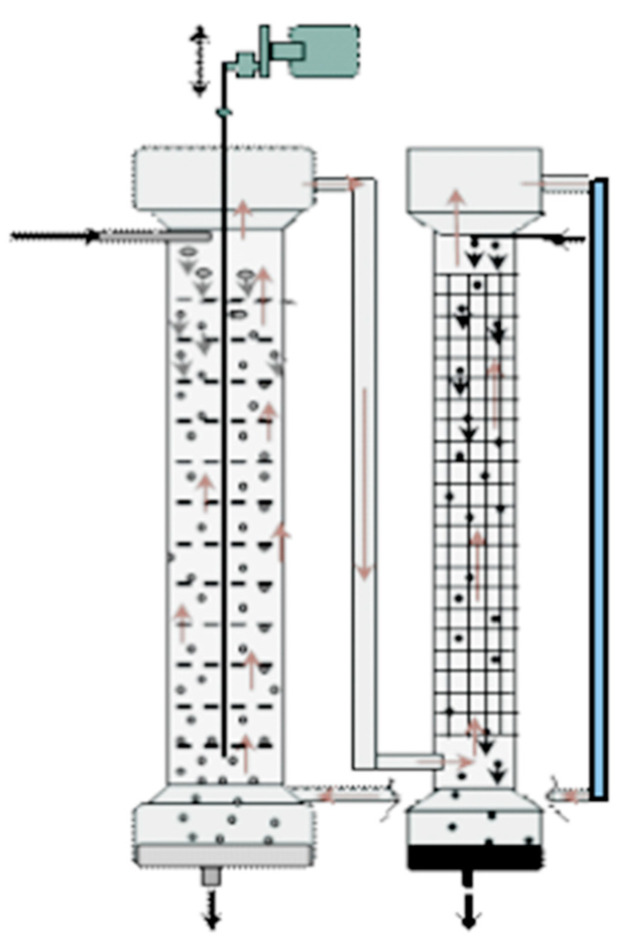
Schematic principles of the operation of the two-column three-phase extraction apparatus with forced circulation of the membrane phase and countercurrent flows of phases in both columns. Red arrows—circulation of the membrane phase; black arrows—flows of the aqueous phases.

The rate of extraction processes in two-column three-phase extraction apparatuses is determined by the rate of mass transfer, the flow regime in the extraction and stripping columns, and the circulation rate of the membrane phase between the columns. With an increase in the rate of mass transfer in the columns and the rate of circulation of the membrane phase between them, the efficiency of three-phase extraction increased. The rate of mass transfer in the columns can be significantly increased by equipping them with vibrating sieve plates. In two-column extractors with convective circulation of the membrane phase, two flow designs, namely cocurrent flows of phases in the extraction column and countercurrent flows of the phases in the stripping column, and vice versa, are possible. For the most efficient extraction separation, the flow directions of the aqueous feed and stripping solutions should be opposite the direction of the membrane phase flow in the extraction and stripping columns. The circulation rate of the membrane phase in a two-column extraction apparatus with convective circulation of the membrane phase is influenced by its configuration (the design of the overflows and the height of the columns). Through the forced circulation of the membrane phase, countercurrent flows of phases can be established in both columns. To organize the countercurrent mode simultaneously in the extraction and stripping columns and to increase the interphase surface, the apparatus shown in Figure 6 is equipped with perforated vibrating discs placed in the extraction column and packing placed in the stripping column. During operation of the vibratory mixer (perforated discs), the aqueous feed phase was crushed into small droplets, and the continuous organic membrane phase was pumped (the direction of circulation is shown by arrows) in the system.

**Figure 7:**
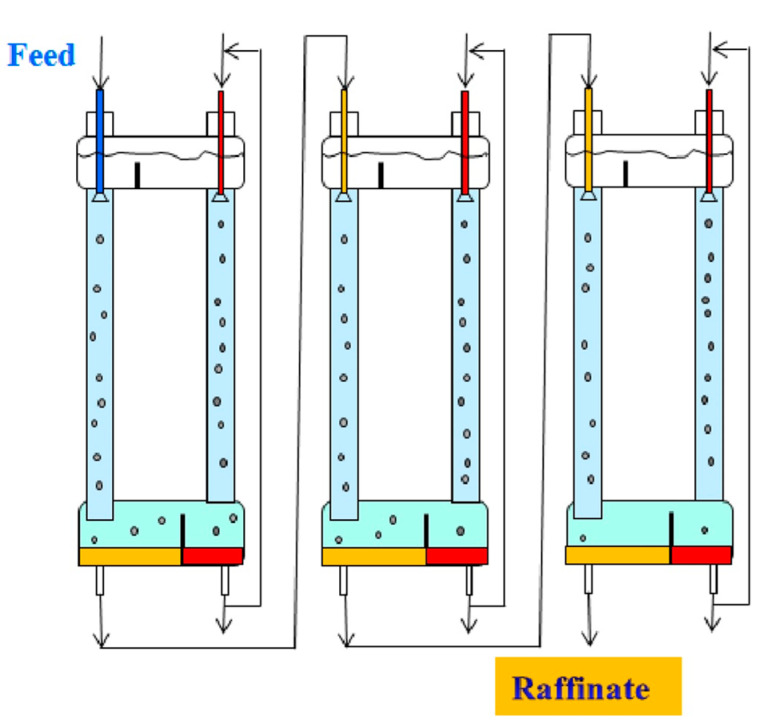
The process of multistage three-phase extraction separation in a cascade of two-column three-phase apparatuses with recirculation of the stripping phase at each stage.

**Figure 8:**
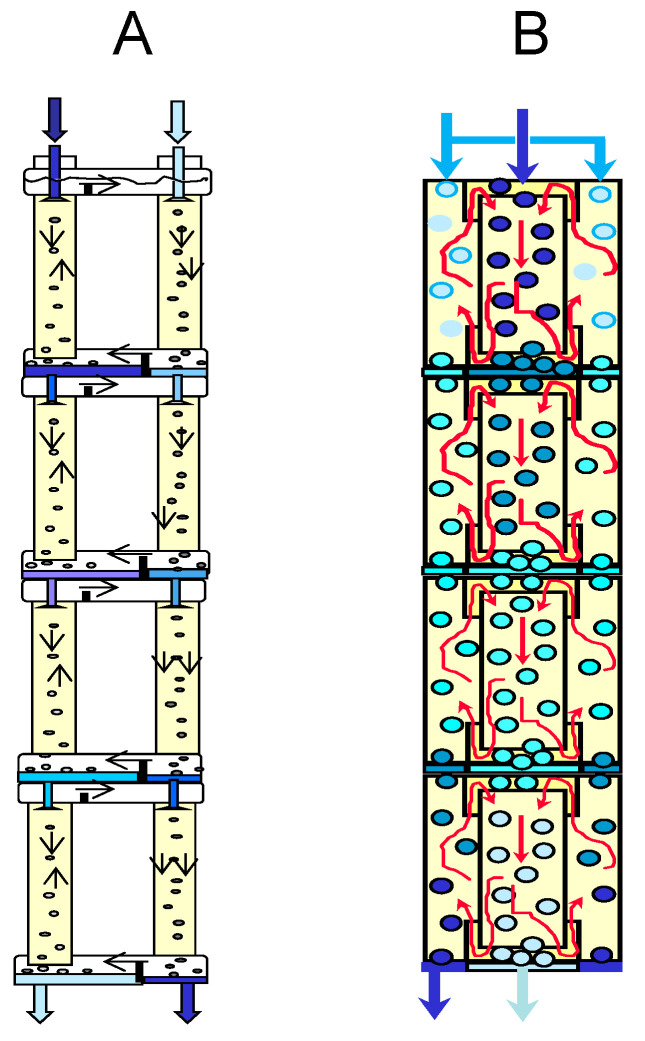
Multistage three-phase extraction apparatus, consisting of two-column stages (**A**) and a multistage two-chamber extraction column (**B**).

An increase in the degree of extraction of metals in two-column three-phase extraction can be achieved using multistage processes that carry out the separation in a cascade of two-column extractors (Figure 7) or in multistage two-chamber columns (Figure 8). In the case when the mass transfer in the stripping column is associated with an irreversible chemical reaction, the efficiency of the separation process is determined by the mass transfer in the extraction column, and it is possible to concentrate the extractable metal in the stripping phase by recycling this phase at the stripping stage. Figure 7 shows an example of such a multistage three-phase extraction separation with recirculation of the stripping phase at each stage.

Figure 8 shows some possible options for a multistage three-phase two-chamber extraction column.

The multistage three-phase extraction separations with the countercurrent flows of phases in extraction and stripping columns can be simulated by Equations (1)–(4). For other phase flow regimes in the extraction and stripping columns, the concentration in the raffinate can be calculated by Equations (1) and (2) using the following Equations for *S*_1_ and *S*_2_ parameters:

Cocurrent flow of the aqueous and organic phases in the extraction column and countercurrent flow of the phases in the stripping column:

(9) $$S_{1}=\frac{1-exp\left[-t_{1}\left(F_{1}+1\right)\right]}{1+F_{1}}\;\;$$

(10) $$S_{2}=\frac{1-exp\left[t_{2}\left(F_{2}-1\right)\right]}{1-F_{2}exp\left[t_{2}\left(F_{2}-1\right)\right]}\;\;$$

Cocurrent flow of the aqueous and organic phases in the stripping column and countercurrent flow of the phases in the extraction column:

(11) $$S_{1}=\frac{1-exp\left[t_{1}\left(F_{1}-1\right)\right]}{1-F_{1}exp\left[t_{1}\left(F_{1}-1\right)\right]}\;\;$$

(12) $$S_{2}=\frac{1-exp\left[-t_{2}\left(F_{2}+1\right)\right]}{1+F_{2}}$$

It should be noted that multistage three-phase extraction is actually a staged version of a supported liquid membrane.

For single-stage apparatuses, Equation (1) reduces to:

(13) $$\frac{x_{1,N}}{x_{1,0}}=\frac{1-F_{1}F_{2}}{{\left[\frac{1-\varphi }{1-\varphi /\left(F_{1}F_{2}\right)}\right]}^{\;}-F_{1}F_{2}}\;\;\;$$

Equation (13) can be used to simulate single-stage three-phase extraction processes and evaluate the influence of the operating parameters on separation efficiency.

The limitations of this study are: (1) The experiments were conducted in three-phase extractors with convective circulation of the membrane phase, which allows two options: countercurrent flow of the contacting phases in the extraction and cocurrent flow of the phases in the stripping columns, and vice versa; and (2) the rate of mass transfer in the extraction and stripping columns was not quantitatively determined, since the rate of circulation of the membrane phase between them was not measured. For further development and industrial implementation of three-phase extraction technology in hydrometallurgy, more detailed studies are needed using single-stage and multistage apparatuses of various designs, including those with forced circulation of the membrane phase. Using single-stage apparatuses, it is possible to evaluate the mass transfer rate in the extraction and stripping stages and determine the influence of the main operating parameters on it. In these experiments, by measuring the circulation rate of the membrane phase, it is possible to determine the rate of mass transfer in the extraction and stripping chambers using Equation (13).

## 4. Conclusions

In this study, the extraction of copper in two-column three-phase extractors with countercurrent flow of contacting phases in the extraction columns and cocurrent flow of phases in the stripping columns was experimentally studied. The 20% solution of LIX-84 in dodecane was used as the organic membrane phase for extracting copper from sulfuric acid solutions. It was shown that the interface area in the extraction column determined the efficiency of copper extraction from sulfuric acid solutions in the apparatuses studied.

The studied devices can be used for wastewater treatment from copper and other harmful impurities. To promote the countercurrent mode simultaneously in the extraction and stripping columns and to increase the degree of extraction of metal ions, it is proposed to equip two-column three-phase extractors with perforated vibrating discs. Multistage processes can be used to further improve the extraction efficiency of the pseudo-liquid membrane method. Mathematical modeling of multistage pseudo-liquid membrane extraction processes was discussed.

## Figures and Tables

**Table 1:** Results of copper extraction and stripping in the system with 20% LIX-84 in decane (equilibrium data).

№	pH_(feed)_	pH_(equilibrium)_	C_Cu(aqueous)_, ppm	C_Cu(organic)_, ppm	Distribution Coefficient
Extraction					
1	3.10	2.11	16.0	2420	151
2	2.96	1.89	21.5	2360	110
3	2.96	1.64	42.5	2580	61
4	2.96	1.55	60	2530	42
Stripping by 20% H_2_SO_4_					
1			10,500	320	33
2			10,750	210	51
3			11,880	200	59
4			12,250	80	153

**Table 2:** Results and conditions of experiments conducted in the small apparatus.

№	Feed Flow Rate, L/h	Distributor Hole Diameters, mm (Extraction)	Stripping Phase Flow Rate, L/h	Distributor Hole Diameters, mm (Stripping)	C_Cu_ (Feed), ppm	C_Cu_ (Raffinate), ppm
1	3.3	2	5.2	2	490	410
2	3.3	2	5.2	2	410	385
3	3.3	0.7	5.2	2	490	355
4	5.2	0.7	8.4	1	490	350
5	3.3	0.7	5.2	1	340	275
6	3.3	0.7	5.2	1	275	222
7	3.3	0.7	5.2	1	2320	2010
8	4.3	0.7	9.6	1	2320	2000
9	5.2	0.7	8.4	1	2320	1960
10	7.1	0.7	10	1	2320	1900

**Table 3:** Results and conditions of experiments conducted in the large apparatus.

№	Feed Flow Rate, L/h	Distributor Hole Diameters, mm (Extraction)	Stripping Phase Flow Rate, L/h	Distributor Hole Diameters, mm (Stripping)	C_Cu_ (Feed), ppm	C_Cu_ (Raffinate), ppm
1	3.3	0.7	5.2	1	460	330
2	5.2	0.7	8.4	1	460	328
3	7.1	0.7	10	1	460	315

**Table 4:** Results of experiments conducted in apparatus 2 when vibrating sieve plates were placed in the extraction column.

№	Feed Flow Rate, L/h	Distributor Hole Diameters, mm (Extraction)	Stripping Phase Flow Rate, L/h	Distributor Hole Diameters, mm (Stripping)	C_Cu_ (Feed), ppm	C_Cu_ (Raffinate), ppm
1	7.1	0.7	10	1	460	20
2	5.2	0.7	5.2	1	525	29
3	5.2	0.7	8.4	1	525	23
4	5.2	0.7	8.4	1	525	2.5
5	5.2	0.7	10	1	525	2.0

## Data Availability

Not applicable.

## Nomenclature

$a_{1}$, $a_{2}$—interfacial area per unit volume of contacting phases in the extraction and stripping columns; $F_{1}=v_{1}/\left(wm_{1}\right)$, $F_{2}=wm_{2}/v_{2}$—extraction and stripping factors; $k_{1}$, $k_{2}$—mass transfer coefficients at the extraction and stripping steps, respectively; *L_1_*, *L_2_*—columns lengths; $t_{1}=a_{1}k_{1}L_{1}/v_{1}$, $t_{2}=a_{2}k_{2}L_{2}/w$—dimensionless parameters characterizing the mass transfer rate in the extraction and stripping columns (the numbers of transfer units); $v_{2}$, $v_{2}$—specific flow rates of the feed and raffinate phases; *w*—specific flow rate (circulation rate) of the membrane phase; $x_{1,0}$, $x_{1,N}$—concentrations of a solute in the feed and in the raffinate.
